# Disentangling the contribution of individual and social learning processes in human advice-taking behavior

**DOI:** 10.1038/s41539-024-00214-0

**Published:** 2024-01-20

**Authors:** Maayan Pereg, Uri Hertz, Ido Ben-Artzi, Nitzan Shahar

**Affiliations:** 1 School of Psychological Sciences, Tel Aviv University, Tel Aviv, Israel; 2 Sagol School of Neuroscience, Tel Aviv University, Tel Aviv, Israel; 3 Minducate Center for the Science of Learning, Sagol School of Neuroscience, Tel Aviv, Israel; 4 Department of Psychology, Achva Academic College, Arugot, Israel; 5 Department of Cognitive Sciences, University of Haifa, Haifa, Israel; 6 Institute of Information Processing and Decision Making, University of Haifa, Haifa, Israel

**Keywords:** Human behaviour, Learning algorithms, Decision

## Abstract

The study of social learning examines how individuals learn from others by means of observation, imitation, or compliance with advice. However, it still remains largely unknown whether social learning processes have a distinct contribution to behavior, independent from non-social trial-and-error learning that often occurs simultaneously. 153 participants completed a reinforcement learning task, where they were asked to make choices to gain rewards. Advice from an artificial teacher was presented in 60% of the trials, allowing us to compare choice behavior with and without advice. Results showed a strong and reliable tendency to follow advice (test-retest reliability ~0.73). Computational modeling suggested a unique contribution of three distinct learning strategies: (a) individual learning (i.e., learning the value of actions, independent of advice), (b) informed advice-taking (i.e., learning the value of following advice), and (c) non-informed advice-taking (i.e., a constant bias to follow advice regardless of outcome history). Comparing artificial and empirical data provided specific behavioral regression signatures to both informed and non-informed advice taking processes. We discuss the theoretical implications of integrating internal and external information during the learning process.

## Introduction

Two forms of learning are predominant in the process of understanding our environment. The first is an unguided trial-and-error learning process, whereby individuals learn from personal experience (henceforth individual learning)^1^. The other form of learning falls under the broad definition of social learning, in which we learn by observing and imitating others, or the main focus of this study - by following explicit advice or instructions^2–4^ (see Lind et al.^5^ for a short discussion on the terminology and diversity concerning social learning). Social learning is considered one of the primary ways by which we learn, especially in humans but also in animals^6^. Despite the distinction between these two forms of learning, it is not simple to decipher and disentangle the unique contribution of each of these processes in explaining human choice behavior. For example, imagine that a friend suggests that you order a particular dish at a restaurant. You might order the advised dish but, from an observer’s perspective, it is impossible to know whether this behavior was due to (a) your own personal prior experience with that type of cuisine irrespective of the advice, (b) the value you assigned to your friend’s advice because her advice panned out before due to her culinary knowledge, or (c) your tendency to comply with advice received from others, regardless of the specific context. Thus, to better understand human choice behavior in following advice, it is critical that we disentangle and estimate the influence of both social and non-social learning processes.

The literature on social learning strategies has dealt extensively with the influence of a single piece of advice on human choice behavior^7,8^. For example, Biele et al.^9^ demonstrated that participants have a general tendency to behave in accordance with a single piece of advice provided to them just one time. In their study, participants were shown to be more likely to choose a card from an advised deck of cards relative to a card deck with a similar positive value but for which they did not receive advice. Biele et al.^9,10^ suggested that individuals have a positive bias when evaluating the outcomes of recommended options relative to options that are not specifically recommended. Several studies support this assumption, having demonstrated that, remarkably, even a single piece of advice or instruction can influence and bias human choice behavior^9–14^. However, it is important to note that most natural environments consist of repeating socially transmitted information, where advice is given more than once, and often by the same individual.

Indeed, previous studies have suggested that individuals tend to keep track of and assess the value of repeating socially transmitted information. For example, one adaptive social learning strategy involves copying the choices of successful individuals, as the knowledge they hold is assumed to be more valuable than that of others^15,16^.

Furthermore, Behrens et al.^17^ found that the associative neural mechanisms that are responsible for processing social information are similar to those at play during individual learning and are subject to updating and learning. Taken together, these studies suggest that agents engage not only in learning the value of their own choices, but also in learning the value of social information. However, it is not clear how these two processes interact and how one might influence the other. For example, Bonawitz et al.^11^ demonstrated that pedagogical teaching (e.g., instructing about the specific use of a toy) limits children’s own exploration when the teacher is assumed to be knowledgeable. However, the same study also showed that children still engaged in exploration when they believed there was more to learn about the toy.

In a related line of research, researchers have explored the specific computational process that underlies individuals’ responses to repeated social advice. For example, Najar et al.^4^ demonstrated that individuals perceive the choices of a demonstrator in a similar manner to how they perceive a positive reward. Hence supposedly, the advice acts as a driving force for behavior. In addition, they showed that only demonstrators who have proven to be knowledgeable are imitated, further supporting the idea that individuals consistently engage in a process of evaluating the information presented to them. Other works were able to demonstrate that the same reward circuits that are activated during individual learning are also activated when receiving advice in a decision-making situation^18^. Moreover, Diaconescu et al.^19^ suggested that people deliberate between social and individual information, while also accounting for the level of certainty or uncertainty they have in the information source. Finally, Rybicki et al.^20^ exhibited support for the assumption that social and individual learning processes rely on similar neurochemical mechanisms (while also indicating the importance of the temporal primacy of the social information^21^). However, these studies used designs that make it difficult to disentangle the influence of different facets of social learning and individual learning on decision-making.

These studies improve our understanding of the processes by which individuals utilize one-time or repeated socially transmitted information when making choices. However, to the best of our knowledge, no study to date has examined whether a bias to follow advice (such as the one that was observed in one-time advice-giving paradigms) remains present in an environment in which advice is repeating. In the current study, we assumed that in a repeating advice-giving environment, wherein individuals are learning the values of different actions (e.g., whether they lead to a positive or a negative outcome), participants will behave in accordance with the advice given for one of three reasons. The first is due to individual learning, which reflects an internal value that was learned in a non-social manner, regardless of whether advice was provided or not. A second option is informed social learning, whereby the individual assigns value to an advised option due to a previous positive experience with the advisor. The third and final option is non-informed social learning, whereby the individual assigns value to complying with advice in general. Whereas individual learning and informed advice-taking often result from trial-and-error learning, non-informed social learning may be due to social norms, such as reciprocity or respect^13,22–24^, or epistemic trust in “advisors”/”teachers”, people who are assumed to have relevant knowledge on which to base their advice^25^.

In the current study, we aimed to disentangle, in a single reinforcement learning task, the influence of advice-taking from individual value-based learning. To do so, we employ a prediction-error-based modeling approach, which was shown to predict and describe human and animal choice behavior across different domains^26^. For example, reinforcement learning has been found to explain algorithmic processes in computational science^27^, simulate and predict human decision-making and learning^28–30^, and shed light on animal behavior^31^. Moreover, the correlation between prediction errors and activity in the human striatum^32^, a key region in the brain’s reward and reinforcement system, underscores the neurobiological relevance of PE-based models in understanding the neural underpinnings of behavior.

**Fig. 1:**
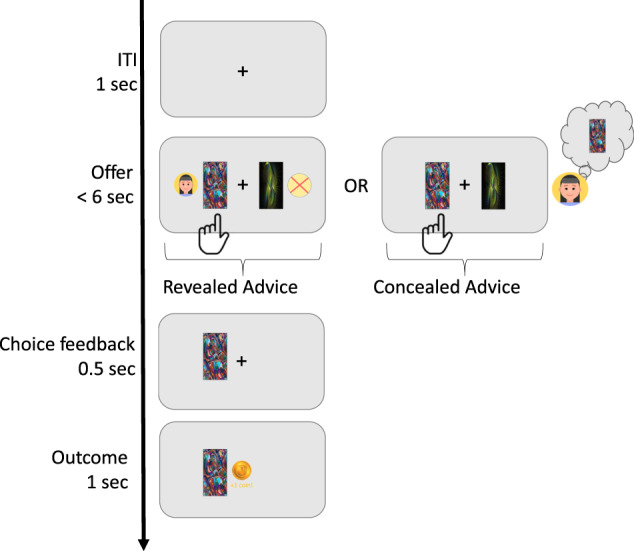
Trial sequence in the student-teacher paradigm. Participants completed a reinforcement learning task, in which two cards were randomly selected and offered to participants on each trial. The cards led to a reward according to a reward probability, unknown to the participant. On each trial, the teacher’s advice was generated by the computer with differing degrees of accuracy. In the revealed advice conditions, the advice was displayed to the participant by presenting the teacher’s avatar next to the advised card (left box in the offer stage). Note that in the revealed advice trials, a red X was presented next to the unadvised card to perceptually balance the cards with a flanking stimulus. Teachers’ choices were also generated during the concealed advice trials, as in the revealed advice trials, but were not presented to the participants. This approach allowed us to examine whether participants’ choices were the same as the teachers’ when the advice was revealed vs. when it was concealed, based on learning and prior experience.

We introduced a novel experimental paradigm in which we randomly interleaved trials with and without advice to allow us to disentangle individual learning from social advice. We estimated individuals’ bias towards following advice in a reinforcement learning task in which advice was either revealed to or concealed from participants. Specifically, participants had to choose between two cards (from a deck of four) that were offered (i.e., a multi-armed bandit task; see Fig. 1). Cards led to monetary rewards ($0 or $1) probabilistically, and participants were asked to make choices to maximize their return. On some of the trials, an artificial teacher gave advice regarding which card should be chosen. Regression analyses demonstrated an influence of the teacher’s advice on participants’ choice behavior, such that participants were more likely to choose the card offered by the teacher when the advice was presented on screen. Furthermore, the tendency to follow advice showed good test-retest reliability, suggesting robustness across two time points. Of main interest in this work, we conducted formal computational models that mimicked the hypothesized cognitive mechanism and tested its fit to the observed data. We found evidence to suggest that participants’ behavior reflect a combination of non-informed social learning, informed social learning, and non-social individual learning. We then conducted analyses that compare simulated with empirical data to identify unique signatures for these processes using dedicated regression analyses. We conclude that individuals have a predominant bias to follow advice when advice from a social agent is repeated and when individual learning occurs. We discuss the theoretical implications of integrating information learned from personal experience with external advice.

## Results

### Analyses overview

**Fig. 2:**
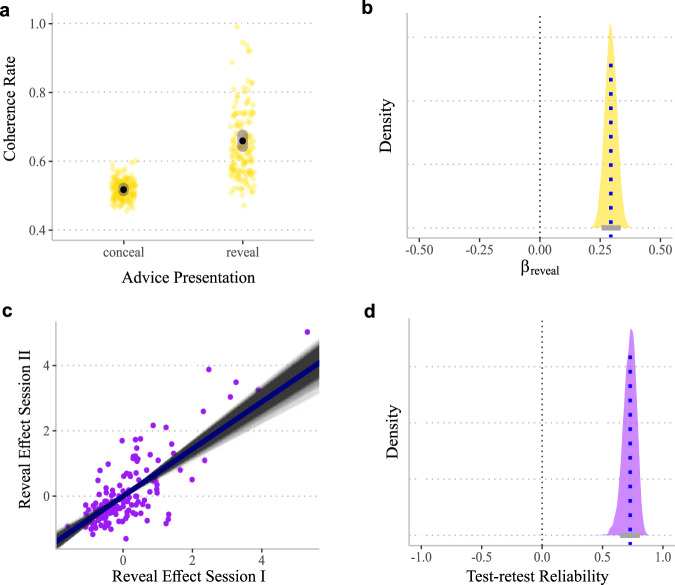
Reveal effect indicating a causal influence of presenting teacher advice on participants’ behavior. We calculated participants’ coherence rates and participants’ tendency to choose the same card as the teacher on trials in which the advice was revealed vs. trials in which the teacher’s choice was concealed. Coherence rates during concealed trials reflect the contribution of individual learning processes to choose the same (high value) card as the one that the teacher recommended. Comparing coherence rates between revealed and concealed trials therefore results in the reveal effect – i.e., the influence of advice on participants choice behavior, above and beyond individual learning processes. **a** Coherence rates as a function of revealing/concealing advice (yellow dots represent the empirical mean coherence levels, black dots represent the mean posterior predictions, and gray ovals represent HDI_89%_). Evidence shows that revealing advice dramatically increased participants’ tendency to choose the same option as the teacher. Thus, advice seems to have a causal effect on participants’ choices, above and beyond any teacher-participant choice alignment. **b** The posterior distribution for presenting the teacher’s advice on the probability to choose the same card as the teacher (gray line indicates HDI_89%_). **c** Test–retest reliability of the reveal effect across two sessions, performed across two adjacent days (posterior median = 0.73). Scatter plot indicates standardized scores for the reveal effect for each session. Gray lines reflect posterior predictive correlations across the whole posterior distribution. **d** Posterior distribution for the test-retest Pearson correlation coefficient.

We examined the contribution of informed and non-informed advice-taking to participants’ choice behavior in a sequential reinforcement learning task (Fig. 1). In this section, we start by establishing a ‘reveal effect’ describing the causal influence of revealing the teacher’s advice to the participant on choice behavior (Fig. 2). This effect uniquely disentangles the influence of advice-taking from trial-by-trial independent learning, which was explored both at the group level and at an individual level (i.e., between sessions test-retest estimates). We then continue to describe the mathematical processes underlying individuals’ choice behavior. Specifically, we used computational modeling to estimate the contribution of three internal latent processes to participants’ choice behavior: (1) individual learning (i.e., learning from experience with the cards, independent of any advice), (2) informed advice-taking (i.e., trial-by-trial learning of the value of following the teacher’s advice), and (3) non-informed advice-taking (i.e., a fixed internal bias to follow advice regardless of choice and outcome history). Finally, we will show further support for the necessity of the two advice-taking processes. We used participants’ estimated computational parameters to simulate data. We independently discouraged participants’ informed or non-informed advice-taking tendencies and demonstrated unique regression signatures for these processes. Overall, we found strong and compelling evidence suggesting a causal effect of advice on participants’ choice behavior, both via informed and non-informed advice-taking processes.

### Theory-independent analysis examining the influence of receiving advice on compliance

We first assessed whether participants demonstrated a bias towards advice-taking across trials and sessions. For this aim, we first calculated a coherence rate as the dependent variable to reflect whether participants chose the same card as the teacher (coded 0/1 when teacher and student selected two different/same cards, respectively), both when the teacher’s choice was revealed and when it was concealed. The coherence rate in the concealed condition served as a baseline, as it reflects the contribution of an individual learning process to the likelihood of agreement with the teacher’s choice, i.e., the likelihood of the participant reaching the same choice as the teacher independently. This is plausible when the participant has learned to identify the more valuable offer, which was often aligned with the choice of the teacher (concealed or revealed). This approach enabled us to examine the unique contribution of the teacher’s advice by comparing coherence rates across the revealed advice and concealed advice conditions, which we termed the reveal effect.

We therefore performed a hierarchical Bayesian logistic regression analysis, predicting participants’ coherence rates as a function of advice presentation (concealed/revealed). We found that coherence rates in the revealed advice condition were higher than the baseline rates in the concealed advice condition, suggesting a causal influence of advice on compliance. Specifically, participants were more likely to choose the card advised by the teacher when the teacher’s advice was revealed (66% coherence rate) compared to when the advice was concealed (48% coherence rate; posterior median estimation for the difference in the population was 0.29, HDI_89%_ = 0.25 to 0.33 probability of direction (pd) ~100% suggesting all of the distribution was positive; Fig. 2a/b].

To further substantiate the estimation of the causal influence of advice on participants’ choice behavior, we examined the test-retest reliability of the tendency to follow advice. Participants performed the task in two separate experimental sessions across two adjacent days (visual stimuli of cards and artificial teachers were changed across blocks and sessions). We performed a hierarchical Bayesian regression analysis and found good test-retest reliability (posterior median = 0.73, HDI_89%_ = 0.64 to 0.81; Fig. 2c/d). Overall, these results demonstrate a strong and reliable causal influence of receiving advice on individuals’ choice behavior in a reinforcement learning task during which advice was given repeatedly. However, these analyses did not allow us to disentangle the effects of informed from non-informed advice-taking. For this purpose, we turned to computational analyses that can explicitly model the contribution of different learning and decision-making processes.

### Computational modeling

We hypothesized that participants’ choice behavior integrates three sources of information: individual learning, informed advice-taking and non-informed advice-taking. We formulated a saturated model that included all three types of information and tested it against three nested models. Specifically, in these models we predicted the agents’ choices based on reward history and experimental conditions. For each trial, we updated the subjective values of the cards (Q-values) based on the prediction error signal^32^. Prediction error is an internal signal by which the agent refines its prediction during the learning and decision-making process. It refers to the discrepancy between the predicted outcome (value or action) and the actual outcome that occurs during the learning process. It plays a crucial role in reinforcement learning as it guides the agent’s learning by indicating how well its predictions align with reality^32–34^. For clarity, we will describe the models we computed. The first model is a baseline model which only had an individual learning component (Model 1). We then added non-informed advice-taking (Model 2) and informed advice-taking (Models 3 and 4) to the models, and lastly, we computed a full model with all three components (Model 5).

### Model 1 (null model)

This model assumes that participants learned the cards’ values from their own experience without considering the presented advice:

1 $${\delta }_{{\rm{chosen}}\_{\rm{card}}}=({\rm{reward}}{\mbox{-}}{{\rm{Q}}}_{{\rm{chosen}}\_{\rm{card}}})$$

2 $${{\rm{Q}}}_{{\rm{chosen\_card}}}={{\rm{Q}}}_{{\rm{chosen\_card}}}+{\rm{\alpha }}* {{\rm{\delta }}}_{{\rm{chosen\_card}}}$$

where α is a learning-rate (free-parameter) and δ_chosen_card_ represents the prediction error for the card selected by the agent (this equation is used in all models). Thus, this model ignores any advice revealed to the participant. In order to choose between the cards, we used a softmax policy:

3 $${\rm{p}}\left({\rm{choice}}\right)=\frac{\exp \left({\beta \cdot Q}_{{chosen\_card}}\right)}{\Sigma \exp \left(\beta \cdot {Qi}\right)}$$

where β is an inverse noise parameter (free parameter), and Q_i_ denotes the Q-values of each card offered in a current trial. Thus, this model had two population-level free parameters (α_chosen_card_, β).

### Model 2 (fixed non-informed advice-taking)

This model is similar to the baseline model only with an additional fixed bias in favor of teacher advice, when it was presented. When the teacher’s advice was presented, action values were calculated according to:

4 $${{\rm{Qnet}}}_{{\rm{advised\_card}}}={{\rm{Q}}}_{{\rm{advised\_card}}}+{\rm{\varphi }}$$

5 $${{\rm{Qnet}}}_{{\rm{unadvised\_card}}}={{\rm{Q}}}_{{\rm{unadvised\_card}}}$$

where φ is a free parameter (unrestricted and could be positive or negative) describing the individual tendency to follow advice regardless of any choice-outcome history during the task.

These Qnet values were then entered into the softmax:

6 $${\rm{p}}({\rm{choice}})=\frac{\exp ({\beta \cdot {Qnet}}_{{chosen}-{card}})}{\Sigma \exp (\beta \cdot {Qne}{t}_{i})}$$

Overall, this model has three population-level free parameters (α_chosen_card_, β, φ).

An additional model (Model 2b) tested dynamic non-informed advice taking, where the bias is moderated by choice difficulty. See Supplementary information for a full report of this model.

### Model 3 (informed advice-taking)

This model assumes that instead of having a general preference for the advised card, participants evaluated the advice during the experimental block via Q-learning. The two options, to follow or not to follow the advice, were updated via Q-learning using a prediction error and learning rate (the same free parameter α from the other models), as shown below (Eqs. (7) and (8)):

7 $${{\rm{\delta }}}_{{\rm{follow\_advice}}}=({\rm{reward}}-{{\rm{Q}}}_{{\rm{follow\_advice}}})$$

8 $${{\rm{Q}}}_{{\rm{follow\_advice}}}={{\rm{Q}}}_{{\rm{follow\_advice}}}+{\rm{\alpha }}* {{\rm{\delta }}}_{{\rm{follow\_advice}}}$$

Next, we calculated Qnet, which incorporated the Q-values of the cards and of following (or not following) advice, and weighted them using ω:

9 $${{\rm{Qnet}}}_{{\rm{advised\_card}}}={\rm{\omega }}* {{\rm{Q}}}_{{\rm{advised\_card}}}+(1-{\rm{\omega }})* {{\rm{Q}}}_{{\rm{follow\_advice}}}$$

We note that the Q-values of the cards were updated using Eqs. (1) and (2), and that the softmax decision function in this model is the same as in Model 2 (Eq. (6)). This model involves 3 free parameters: learning rate for the cards and for following advice (α), inverse noise parameter (β), and weighing between the Q-values of the cards and of following advice (ω).

### Model 4 (moderated informed advice-taking)

In the previous model, participants were assumed to update the value of following advice based on the observed outcome. However, the outcome (reward vs. unrewarded) is not only a function of how accurate the teacher was, but also depends on the expected value of the selected card. Participants might thus moderate their value update for the teacher’s advice based on their current expectation for a reward given a specific chosen card. We therefore included a model that also includes a moderation of the Q-values for following advice as a function of the prediction error for the chosen card (see Eq. (1)). According to this model, updating the value for following advice is a function of both the observed outcome and the Q-values of the specific choice at hand. Hence, if the prediction error for the cards is relatively small (in absolute values) based on individual learning, less updating of the value of the teacher is required, and vice versa. To update of the Q-value to follow (or not follow) advice, Eq. (7) was replaced by Eq. (10), as follows:

10 $${{\rm{Q}}}_{{\rm{follow\_advice}}}={{\rm{Q}}}_{{\rm{follow\_advice}}}+{\rm{\alpha }}* {{\rm{\delta }}}_{{\rm{follow\_advice}}}* {\rm{|}}{{\rm{\delta }}}_{{\rm{chosen\_card}}}{\rm{|}}$$

This model involves the same three free parameters as the previous model: learning rate (α), inverse noise parameter (β), and weighing between the Q-values of the cards and of following advice (ω).

### Model 5 (informed and non-informed advice-taking)

This model combines Models 2 and 3 and assumes both a general preference for the advised card, and an evaluation of the teacher through the reward history (Eq. (11)).

11 $${{\rm{Qnet}}}_{{\rm{advised\_card}}}={\rm{\omega }}* {{\rm{Q}}}_{{\rm{advised\_card}}}+(1-{\rm{\omega }})* {{\rm{Q}}}_{{\rm{follow\_advice}}}+{\rm{\varphi }}$$

### Model fitting

We performed a model comparison using a leave-one-block-out approach, as described in the Method section. We calculated a difference distribution for each paired model comparison and estimated the expected log probability density (elpd) difference and the standard error of the difference distribution (using ‘loo’ package; with an elpd difference of 2 times the standard-error considered substantial^35^). We found Model 5 to be the winning model (see Table 1). Parameter recovery is depicted in Supplementary Information.

**Table 1:** Model comparison results – winning model compared to other models.

Model	Expected log probability difference compared to the winning model (Model 5 – informed and non-informed advice-taking)
Null model	−4147.2 (88.4)
Model 2 (fixed bias)	−514.1 (33.4)
Model 3 (teacher evaluation)	−701.3 (48.8)
Model 4 (moderated teacher evaluation)	−490.7 (47.0)

Elpd (expected log probability density) was calculated using a leave-one-(block)-out cross-validation approach. An elpd difference that is larger than 4 and at least twice the standard error is considered to be significant evidence for the winning model^35^.

Elpd difference standard errors are noted in brackets.

### Estimated parameters

**Fig. 3:**
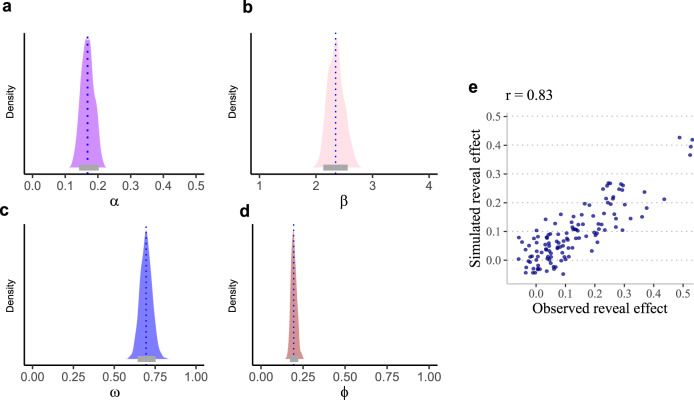
Estimated parameters for the best fitting model. Posterior distribution of the winning model parameters (**a**–**d**): The fixed effect posterior distribution of the learning rate (α, panel **a**), inverse temperature (β, panel **b**), informed advice-taking (ω, panel **c**) and non-informed advice-taking bias (φ, panel **d**). Overall, results suggest that participants clearly engaged in both types of advice-taking. This can be seen in an ω population parameter centered at ~0.70 (1 represents only individual learning with no informed advice-taking, 0 represents only informed advice-taking with no individual learning). Furthermore, the φ parameter suggests a positive tendency to follow advice regardless of any task experience (0 represents no non-informed advice-taking, a negative value indicates a tendency to not choose advised cards). The blue dashed line represents median values, the gray horizontal bar represents 89% CI. **e** To create a visual illustration of our model’s ability to capture choice behavior, we simulated artificial data based on the empirical data and individuals’ parameter estimation. We then calculated and plotted the ‘reveal effect’ from empirical data and artificial data for each subject. The scatter plot clearly indicates an excellent fit, such that the individuals’ estimated parameters generated a very similar reveal effect to the one observed in the empirical data.

The estimated parameters for the winning model highlight the contribution of individual learning, informed advice-taking, and non-informed advice-taking (Fig. 3). The ω parameter was estimated to be ~0.70 (Fig. 3c), indicating a reliance on individual learning, but also on informed advice-taking, which tracked the accuracy of the teacher. This value indicates that a highly accurate teacher could influence the decisions made by participants beyond individual learning, a finding that is in line with the reveal effect discussed above. Parameter φ was estimated to be ~0.2 (Fig. 3d), indicating a tendency to follow advice in a manner that is unrelated to the teacher’s accuracy. This fixed contribution is the basis of non-informed compliance and contributed to the reveal effect as well. This tendency increased the likelihood of following advice even when it went against one’s individual experience and/or when the teacher’s accuracy was low.

### Associations between the winning model and empirical data

To demonstrate the association between the winning model parameters and the model-agnostic results, we estimated the individual bias to follow advice for each participant and for each simulated agent. To do so, we simulated artificial data with the same number of trials as the empirical data using individuals’ parameter estimation (we used the mean posterior for each individual and parameter). We then calculated and plotted the ‘reveal effect’ from empirical data and artificial data for each subject based on the model and examined the correlation between them. We found a strong positive correlation (Pearson *r* = 0.83, pd~100%; CI_89%_ = 0.77–0.86; Fig. 3e), showing that the model successfully replicated the behavioral results.

### Behavioral signatures for the winning model

Thus far, our modeling results (Table 1) show clear evidence suggesting that individuals are using both informed and non-informed advice-taking mechanisms. Specifically, elpd suggested that using both informed and non-informed processes substantially increased our ability to predict left-out blocks. To illustrate the existence of both types of advice-taking in the empirical behavioral data, we additionally performed a set of analyses that compare simulated results based on the winning computational model with the behavioral data. We first simulated two data sets for each individual, based on the individual empirical parameters’ estimation that was gained from the winning model. This set of analyses serves as two private cases of Model 5: One in which ω = 1 (and so only non-informed advice-taking takes place here); and the second in which φ = 0 (and thus only informed advice taking takes place in this dataset). In the first set (non-informed advice-taking), we fixated the ω parameter (i.e., ω was set to 1), thus forming a simulation by which only non-informed advice-taking underlies the learning process. In the second data set, (informed advice-taking) we muted the φ parameters (i.e., φ was set to 0), thus forming simulated data in which only the informed advice-taking underlies the learning process, and there is no bias to follow advice. This allowed us to examine the reveal effect in datasets that were artificially constructed using only one of the advice-taking processes at a time, and compare them to the empirical behavioral data. Therefore, these analyses were constructed in order to show behavioral signatures for both learning processes that constitute the winning computational model: non-informed and informed advice-taking.

#### Signature for non-informed advice-taking

**Fig. 4:**
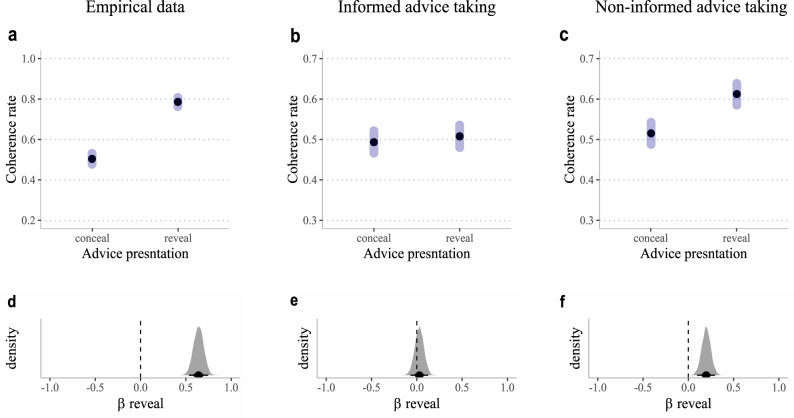
Results of simulation analyses showing distinct regression signatures for non-informed advice-taking and posterior distributions. We analyzed three data sets: (1) empirical data, (2) artificial informed advice-taking (simulated using individuals’ estimated parameters after muting the non-informed advice-taking by setting φ = 0 across subjects), and (3) artificial non-informed advice-taking (simulated using individuals’ estimated parameters after discouraging the informed advice-taking process by setting ω = 1 across subjects). The ‘reveal effect’ calculated only from two trials per block and per subject; the first trial in which advice was revealed and the first trial in which advice was concealed. Thus, informed advice-taking should not be able to produce a reveal effect in this analysis. As hypothesized, we found a substantial reveal effect in the empirical data (panel **a**, and its posterior distribution in panel **d**), which was mimicked only by the non-informed artificial data (panel **c**, and its posterior distribution in panel **f**), but not the informed artificial data (panel **b**, and its posterior distribution in panel **e**).

We calculated the ‘reveal effect’ using only two trials for each participant, per block – the first time a conceal trial was presented and the first time a reveal trial was presented. We reasoned that during such an early stage in the task, informed advice-taking would not be able to produce a reveal effect. Note that each block included a novel teacher, with whom the participant did not have prior experience. For the empirical data, we found a substantial reveal effect (median = 0.64, CI_89%_ between 0.555 to 0.73, pd ~100%; see Fig. 4, panels a, d). We found a similar effect in the non-informed artificial dataset (median = 0.20, CI_89%_ between 0.12 to 0.28, pd ~100%; see Fig. 4, panels c, f). Importantly, we found evidence against a reveal effect for the complementary informed advice-taking artificial data set (median = −0.03, CI_89%_ between −0.0555 to 0.11, pd = 74.40%; see Fig. 4, panels b, e).

#### Signature for informed advice-taking

To capture a unique signature for informed advice-taking, we examined the influence of outcome on choice behavior. Specifically, we calculated a coherence repeat rate dependent variable, which reflected whether participants exhibited the same behavior (following the teacher’s advice or not) across two consecutive trials, *n* and *n* + 1 (coded 0/1 for different/same behavior, respectively). We then used Bayesian logistic regression analyses to predict coherence repeat as a function of previous outcome (rewarded vs. unrewarded) and advice presentation (concealed vs. revealed on both *n* and *n* + 1 trials), as well as their paired interaction. Note that we did not include trials in which advice presentation was different across n and *n* + 1 trials to allow for a more precise comparison. We reasoned that the interaction of previous outcome X advice presentation would be present only for informed advice-taking. Specifically, when advice is presented, a reward on trial *n* should increase coherence on trial *n* + 1 compared to unrewarded n trials (as can be seen in the updating of the Q_follow_advice_ value in the winning Model 5). However, in concealed advice trials, the value for taking/rejecting advice should not be updated, and so the reward on trial n should not affect coherence rates. Note that we also added teacher accuracy in the *n*^th^ trial as a fixed effect only with no interaction, allowing us to control for overall coherence baseline rates, which might change with teacher accuracy.

**Fig. 5:**
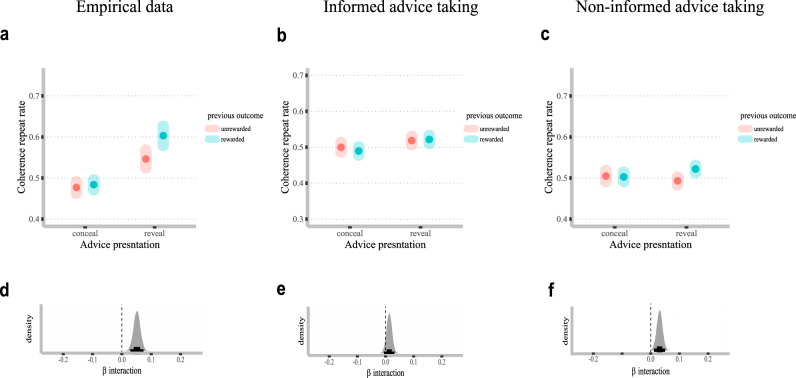
Results of simulation analyses showing distinct regression signatures for informed advice-taking and posterior distributions. We analyzed three data sets: (1) empirical data, (2) artificial informed advice-taking (simulated using individuals’ estimated parameters after muting the non-informed advice-taking by setting φ = 0 across subjects), and (3) artificial non-informed advice-taking (simulated using individuals’ estimated parameters after discouraging the informed advice-taking process by setting ω = 1 across subjects). We estimated the likelihood of observing a repetition in coherence from trial *n* to *n* + 1 as a function of outcome in trial n (unrewarded vs. rewarded) and advice presentation (concealed vs. revealed on both trials). We found a significant paired interaction suggesting that previous reward increased coherence repetition, only for trials in which the teacher’s advice was presented (panel **a**, and its posterior distribution in panel **d**). This suggests that participants assigned value to teacher’s advice as a function of the observed outcome. Importantly, this interaction was mimicked only by the informed advice-taking artificial data (panel **b**, and its posterior distribution in panel **e**) and not the non-informed advice-taking artificial data (panel **c**, and its posterior distribution in panel **f**). These results clearly mirror our model comparison results, suggesting that participants engaged in both informed and non-informed advice-taking.

For the empirical data, we found evidence in favor of a previous outcome X advice presentation paired interaction (median = 0.05, CI_89%_ = 0.03–0.07, pd~100%; Fig. 5, Panels a, d). This suggests, as we predicted, higher coherence repeat rates after a rewarded vs. unrewarded n trial, but only when advice was revealed. When the advice was concealed, the outcome of trial n had no influence on coherence repeat rates. A similar (but smaller) positive interaction was found for the informed artificial data (in which non-informed advice-taking was lesioned; median = 0.03, CI_89%_ = 0.01–0.0, pd = 99.85%; Fig. 5, panels b, e). Importantly, the non-informed artificial data set (in which informed advice-taking was halted) did not show evidence in favor of a previous outcome X advice presentation paired interaction (median = 0.01, CI_89%_ = 0 to 0.03, pd = 91.55%; Fig. 5, panels c, f).

Overall, these analyses were able to show two unique regression signatures: one that is predicted only by informed, but not non-informed advice-taking and one that is predicted only by non-informed but not informed advice-taking. Importantly, both effects were observed in the empirical data set, providing further evidence for our model comparison conclusion which suggests that both types of advice-taking are required to explain the data.

Yet, one might argue that these data could have been gained due to non-social aspects of the task as well (such as demand characteristics). To control for this alternative explanation, we also tested a non-social version of the student-teacher task, in which we replaced the virtual teacher with a lottery wheel. The experimental procedure was the same, except that the virtual teachers were replaced with a non-human lottery wheel (see Supplementary information). The results indicated a smaller reveal effect in the non-social experiment (i.e., increased tendency to choose the advised card in trials where the advice was revealed vs. concealed), suggesting that advice that is symbolically framed as social information holds a greater causal influence on participants’ tendency to follow advice.

## Discussion

Previous studies have demonstrated that learners integrate social information into their value-based decision-making. However, one prominent unresolved issue with prior studies concerns the inability to separate unguided internal decisions (individual learning) from decisions based on external advice. Moreover, we argue that following advice could be further differentiated into informed advice-taking (learning the value of following advice), and non-informed advice-taking (bias to follow advice regardless of an advisor’s previous accuracy). In the current study, we therefore opted to approach these two issues using a reinforcement learning design comprising repeated advice. With this experimental design, we were able to disentangle individual from social-based decisions, while also investigating the reliability of advice-taking behavior.

The results showed that, behaviorally, participants demonstrated a robust bias towards following advice, which was a rather stable bias across separate measurements. Computational modeling enabled us to identify the underlying mechanisms and showed that the results are likely due to both non-informed advice-taking (i.e., a fixed bias towards following advice), and informed advice-taking (i.e., learning the value of following the teacher’s advice). Analysis of the empirical data showed two complementary findings for the non-informed and informed processes, respectively: (1) participants demonstrated a bias to follow advice even on their very first encounter with the teacher; and (2) reward history had a causal effect on future advice-taking behavior. Signatures from simulations analyses comparing artificial and empirical data showed a double dissociation supporting the necessity of considering both processes in order to explain the empirical data. Taken together, the results suggest that an intelligent process occurs in which teachers’ outcome history (i.e., accuracy) contributes to participants’ tendency to follow their advice.

It should be noted that in this work, we aim to study processes involved in following advice during social learning, which are considered to be different than processes involved in other forms of social learning, such as observational learning. For example, it was shown that in observational learning there are separate prediction error mechanisms involved for actions and outcomes at both the behavioral and neural level^33^. However, observational learning often involves observing not only the choices, but also the resulting outcomes; whereas advice is different, since the information is intentionally transmitted to the learner usually without incurring personal costs on the advisor, and thus these differential prediction processes might not be relevant in advice taking. In support of this dissociation, Hertz et al.^13^ showed that participants deliberated less and were more willing to follow advice than to copy the choices of another player. That study also indicated that following advice is related to trust in others, thus raising the issue of the role that the intentions of the advisor play in this type of learning^36^. Therefore, we note that the results gained in the current study do not necessarily expand to other forms of social learning that were not explicitly examined here.

The computational mechanism found in the current study resembles that of Diaconescu et al.^19^, which showed that people have a social bias towards following advice, accompanied by an arbitration between social and individual sources of information. In that study, participants played a lottery game where they could base their predictions for the raffle’s results based on their individual experience and/or on advice. Importantly, the advisor’s intention to help varied throughout the experiment, such that participants were hypothesized to adjust the source of information that they rely on based on this volatility (i.e., based on the perceived precision of each source of information). Using a Hierarchical Bayesian model, they showed that participants tracked the volatility of each information source and arbitrated between them, taking into consideration the advisor’s precision and stability. The current work affirms and extends their findings by showing that a simple experimental manipulation can disentangle the causal effect of advice from individual learning. That is, by measuring the difference in coherence rate for trials where advice was revealed vs. concealed, we can measure the causal effect of advice on choice behavior in a continuous learning paradigm, without having to use computational modeling. Furthermore, our RL modeling supported Diaconescu et al.^21^ showing that indeed individuals tend to engage in both individual learning and advice-taking. We further show that this is true in a much less volatile environment and suggest that both informed and non-informed types of learning affect the value of the individual to follow advice.

Najar et al.^4^ also showed that the demonstrator’s choices directly influenced those of the player, but it was shown in an imitation process such that the players mimicked the previous choices of the demonstrator. Najar et al.’s^4^ winning value-shaping model allows for long-lasting learning of social inputs, but importantly, similar to Diaconescu et al.’s^19^ model, the socially oriented bias towards “advised” options is bound with the specific choice made by the demonstrator. Hence, we suggest that the bias parameter in our models (i.e., non-informed advice-taking) might capture a phenomenon that relates to the normative aspect of following others’ advice since it demonstrates a general bias that is not limited to a specific choice (see Mahmoodi et al.^24^ for a study concerning the involvement of normative and informational conformity in change of mind).

One explanation for a bias towards following advice comes from the work of Biele et al.^10^, which showed that following advice was intrinsically rewarding in and of itself, thus explaining the long-lasting influence of a single piece of advice provided one time. Moreover, Doll et al.’s^12^ work supported a computational model by which learners favor advice over individually-learned information, even when the advice was incorrect, and in Najar et al.’s^4^ model, the demonstrator’s choice itself serves as a pseudo-reward in the computational mechanism. Presumably, such a bias could rely on epistemic trust, which represents a “trust in knowledge”, or a tendency to evaluate new social information as accurate and reliable and to integrate it into one’s own learning environment^37,38^. Epistemic trust has been extensively studied in young children^39–41^, and studies show that children are able to evaluate their informants during learning, and estimate whether they are knowledgeable and trustworthy. For example, Woo and Spelke^42^ showed that even toddlers are able to both learn from the outcome of an agent’s behavior as well as to evaluate the intention of the social agent. A unified framework by Eaves and Shafto^37^ suggests that epistemic trust complements pedagogical inference, where informants are *assumed* to be trustworthy. By integrating the results of these studies, we can conclude that adaptive learning results from a combination of having trust in, and evaluating, a pedagogical agent. A social learning strategy such as “copy when model successful” allows for this type of evaluation of the informant^43^. Finally, trust has been shown to influence learning from advice, but not from observation^13^; for example, one study showed that participants with high levels of paranoia were less likely to comply with advice, but did not differ from individuals with low paranoia levels in the extent to which they copied an observed decision. It seems that both processes are mirrored in the current results, which demonstrate a general bias to trust the advisor and follow advice (non-informed) and form an evaluation of the teacher (informed advice-taking).

The current results also relate to other works concerning social learning across different areas. For example, one line of research aims to theoretically distinguish between different avenues through which one learns from others’ behavior. It was found that, in young children, learning is often the result of “blind imitation”, such that by simply copying others, one learns new behaviors^44^. In contrast to imitation, another form of social learning is emulation, which is considered more resource-consuming and involves the learning of the effects or goals of the actions (as opposed to the actions themselves). A neuro-computational study concerning imitation and emulation learning processes demonstrated that brain networks employ a highly adaptive and dynamic approach to allocate control between choice imitation and goal emulation, depending on emulation reliability, or the relative uncertainty in each strategy’s predictions^45^. In the current study, participants could have followed the teacher’s selection by either blind imitation or emulation – i.e., copying the teacher’s choices in order to maximize rewards. However, the interaction between a fixed bias to follow advice and an observed pattern in which participants learned from the outcome history goes against a “blind imitation” approach, suggesting that participants deliberated the value of following advice and then made a choice of whether to emulate the teacher’s behavior or not. This is supported in the simulation analyses showing the effect of the informed advice-taking process. Nonetheless, the robustness of the bias to follow advice also suggests that participants utilized both processes when making their choices, such that the bias itself could be considered blind imitation.

Thus, one question that comes to mind concerns the boundary conditions for such a strong bias to follow advice. It should be noted that the bias to follow the teacher’s advice is highly related to what is termed informational conformity - i.e., the tendency to conform with social information in uncertain/unknown environments, where we believe that others hold valuable information^46^. Indeed, previous works have shown that individuals have a higher tendency to use social information when it is harder to reach a decision on their own, or when they are less confident in their own choice^47^. In the current study, we used a probabilistic reinforcement learning task in which the uncertainty level was high due to the shifting values of the cards across trials, which also required that participants keep track of the outcome history per card. This was done in order to increase learning demands^48^. Hence, it is possible that a different (perhaps weaker) bias towards following advice would have been found with a different design in which the learning demands were lower.

This study holds several limitations. The first involves a broad question regarding the “social” nature of the task, given that participants were not deceived to think that an online human agent was behind the virtual teachers. As per definitions, we refer to social learning under a broad classification that concerns following/learning from signals that originate from other agents. In contemporary naturalistic human social settings, individuals are consistently subjected to social content that is circulated by anonymous virtual entities (e.g., online reviews, social navigation applications, and other similar social platforms). We would like to propose that the frequent exposure of participants to such anonymous entities in social platforms, increases the likelihood that participants will address symbolic virtual agents in empirical studies (such as a virtual teacher in the current study) as social information^49,50^. This view is in line with many other studies that conveyed information to participants using different illustrations, images, and text (either symbolic or explicit) to study social learning processes^13,49–52^. Specifically, Vélez and Gweon^50^ used a card game and incorporated advice from an artificial agent to test how people integrate partial individual information with incomplete advice. In another work, Atlas et al.^52^ examined fear conditioning to test whether instructed knowledge modulates feedback-driven learning. The social information was conveyed through emotionally charged faces and verbal instructions to issue fear conditioning. Moreover, in previous works, such minimal instructions were enough to elicit social behavior online that was similar to social behavior in the lab under more elaborate social settings^53^ and even to induce online participants to sacrifice some payment to gain social rewards^54^.

In search for further empirical support to the claim the virtual teachers were perceived as providing social information, we performed an additional experiment using a non-social cue, and found that when the advisor is framed as a lottery wheel, rather than a virtual teacher, the reveal effect diminishes (see Supplementary Information). This is in line with previous studies^13^, who found a small but significantly greater tendency to follow advice than to mimic observed actions by others. Therefore, an advantage towards following advice (over observation)^13^, taken together with the advantage we found for following advice (over the lottery wheel) – might indicate that there is an additional meaning for advice that is symbolically framed as social information. However, more studies should be designed to directly examine this question, perhaps as a matter of different social framings, ranging from overtly non-social to the presence of another peer that provides advice.

Two methodological limitations concern the experimental design and the computational modeling approach. We chose an experimental design that will suit repeated interactions with the card game and the virtual teachers. Given that we aimed to measure not only the bias towards the advice, but also the learning processes concerning both the cards and the teachers, the blocks needed to include a rather large number of trials that will enable a reliable measure. However, as opposed to other studies that used a single advice, we had to keep participants on their toes and encourage learning throughout the block, and so both the cards’ expected values and the teachers’ accuracy gently drifted during the block (e.g., Daw^55^). Overall, the accuracy level of the teachers was above 50% at all times, and with no sudden changes. Also, we did find that the value of taking advice in the winning model followed the teacher’s accuracy across trials (see Supplementary Information). However, overall, the current study was mostly designed to allow a reliable measure of the interaction with the teachers, rather than testing whether there were major alterations in the participants’ responses to the teacher’s advice. This issue should be further tested in future studies with a more deliberate experimental design.

Regarding the computational modeling approach, we exclusively examined a prediction-error-based reinforcement learning model to elucidate the learning processes. While this approach offers valuable insights into how agents update their beliefs and actions in response to prediction errors, it is important to acknowledge the existence of alternative decision-making frameworks that were not explored in this study. One option could be active inference, which focuses on minimizing surprise by iteratively updating beliefs and selecting actions that bring sensory inputs into alignment with their internal generative models^56^. Another alternative could be forward models that operate by generating predictions of sensory outcomes based on an agent’s planned actions^57^. Each of these alternative approaches offers distinct perspectives on decision-making, and future research may benefit from investigating their applicability to our research domain.

Moreover, the current findings demonstrate strong evidence for a mixture model where two learning mechanisms are taking place simultaneously: non-informed and informed advice-taking. The combined model (Model 5) involving both processes was constructed by integrating the basic formulation of each of these mechanisms. Specifically, in the winning mixture model (Model 5) non-informed advice-taking was formulated as a value bonus assigned to the instructed card, and informed advice-taking was updated based on the observed outcome. It is of course possible that a more complex mixture process takes place during this type of learning. For example, the moderated informed advice-taking mechanism described in Model 4 might even further improve the predictive accuracy of the empirical data if combined within a mixture, informed and non-informed, model. Further studies are thus required to provide a more comprehensive examination of different informed learning mechanisms that might subside with the non-informed advice-taking tendencies.

Future studies should also look further into the conditions that lead to such a strong bias. As suggested, one thing that comes to mind is the influence of decision difficulty. Second, the current experimental paradigm, which involves repeated interactions with an advisor, is a good candidate for examining how people evaluate the value of the advisor and whether and how the advice accuracy influences behavior throughout the learning process. Although we found a positive correlation between the internal values of following advice and the teachers’ accuracy rates (see Supplementary Information), this is not the main focus of the current study. Future studies could also examine this issue by formally modeling this type of learning, for example by using a full Bayesian updating of the teacher’s accuracy rates.

To conclude, in this work, we were able to disentangle two types of social learning: informed and non-informed advice-taking. Our computational model supports the involvement of both processes, alongside individual learning. Finally, advice-taking was shown to be reliable across measurements, thus behaving as a trait-like decision bias to follow external advice.

## Methods

### Participants

We recruited participants through the Prolific online platform for a two-part experiment in return for monetary compensation. One hundred and sixty-six Prolific workers completed Session 1, and 153 completed Session 2. The final sample comprised 153 participants (mean age = 26.63, range 18 to 46; 90 men, 59 women, 4 other gender identification). Participants reported normal or corrected vision and no current or past psychiatric or neurological diagnosis. The study protocol was approved by the Research Ethics Council of Tel Aviv University and all participants signed an informed consent form before participating in the study.

### The student-teacher task

Participants completed two sessions of a multi-armed bandit reinforcement learning task during which they were asked to choose between cards in order to gain rewards (Fig. 1). The task included four cards, and in each trial, the computer randomly selected and offered two cards for participants to choose from. Each card led to a reward according to a reward probability that changed gradually across trials (the temporal profile of reward probabilities was generated using a noisy random walk; see Supplementary Fig. 1 in Supplementary Information). Participants were informed regarding the drifting-value nature of the cards (i.e., the cards’ changing value) and were instructed to try to do their best to gain as many rewards as possible, which would be converted to a monetary bonus at the end of the experiment. In addition, the task included avatars of teachers, that latently chose a card on each trial based on a noisy estimation of the cards’ true expected values (see Supplementary Information). During the instructions phase, we informed participants about “virtual teachers” that will give them advice from time to time and suggest which of the cards should be chosen. The “teachers” were represented by small illustrations of different faces (see Fig. 1 for an example). Participants were instructed that the virtual teachers were familiar with the card game, but do not know whether a card will produce a reward, and so it is up to the participant to decide whether to follow their advice. Note that no deception was used, the participants knew that the teachers were virtual and designed by the experimenter. Participants were familiarized with the teacher’s avatar at the beginning of each block and were told that the teacher would direct them from time to time, to help them choose the best card. In practice, the virtual teacher had access to the true expected value of the cards, and made a decision using a noisy softmax (see Supplementary information). To encourage participants to keep track of both the cards’ value and the teacher’s advice, we slowly and randomly changed the noise in the teacher’s decision (changing the softmax noise parameter using a slowly drifting random walk, see Supplementary Information). On average the teacher accuracy was 63% and ranged between 53 to 77% accuracy.

An additional important detail is that the teachers’ choices were generated by the computer on all trials but revealed to participants as advice only on 60% of the trials. The rate at which the advice was revealed was chosen based on an assessment of the required number of trials per condition. Hence, the task involved two primary conditions, concealed advice and revealed advice, which alternated randomly between trials. In “concealed advice” trials, participants were only presented with the two offered cards, and did not observe the teacher’s choice. In the “revealed advice” trials, the teacher’s choice was revealed to the participants (Fig. 1). To indicate the advice, the teacher’s avatar appeared next to the advised card, and a red X appeared next to the other card for perceptual balance. Finally, to encourage participants to keep track on both the cards’ value and the teacher advice, we slowly and randomly changed the teachers ability to pick the more valuable card (using a random walk, see Supplementary Information). The teachers’ accuracy was based on the Softmax function, such that the *β* component followed a stochastic distribution (see Supplementary Fig. 2 for teachers’ *β*, Supplementary Information). The Softmax for teacher accuracy is provided in Eq. 12.

12 $${\rm{p}}({\rm{i}})=\frac{\exp \left.\left(\beta \left(t\right)\times p\left({rw},i\right)\right)\right)}{\mathop{\sum }\nolimits_{n=1}^{2}\exp \left.\left(\beta \left(t\right)\right)\times p\left({rw},i\right)\right)}$$

The probability of the teacher choosing card *i* is a function of the *β* of the teacher on a certain trial *t*, and the true probability of that card *i* to lead to a reward.

At the beginning of the session, participants were presented with task instructions, as well as a multiple-choice quiz which they had to complete with 100% accuracy in order to continue to the card game. Participants completed the student-teacher task across two sessions (one day after the other), and at the end of the experiment, were paid a fixed amount (£2.5 per session) plus a bonus [£0.75 per session, if they completed both sessions (range: £0-£1.5, mean = £0.75)]. Each session involved three blocks with 130 trials each, and a short break was provided after 65 trials. Each block introduced a new set of visual stimuli - the cards and the avatar teachers were replaced on each block in every session. Each trial began with a 500 ms fixation, after which the cards (with or without the teacher’s advice) appeared until a choice was made or until 6 seconds had elapsed. After participants made their choice, the chosen card appeared on the screen for 500 ms, and the reward feedback was displayed alongside the selected card for 1000 ms. Finally, a black screen appeared for 500 ms before the next trial started (Fig. 1).

### Preprocessing

Preprocessing involved locating data points during which the participant was unengaged with the task: Participants who kept pressing the same key (key repetition on more than 90% of trials) were set for exclusion, yet none reached this criterion (0 participants excluded). Trials with unreasonably fast or slow reaction times (<200 ms or >4000 ms) were omitted (resulting in 3.75% of all trials). Participants with more than 25% excluded trials due to fast/slow reaction times (13 participants) were excluded from the analyses altogether. The remaining data set included 140 participants, with an average of 749 trials per subject, resulting in a total of 104,918 observations that were later used for Bayesian parameters updating in the main analysis.

### Bayesian parameters estimation

We performed Bayesian logistic regression and reinforcement learning computational modeling analyses using ‘brms’, ‘rstan’, and ‘loo’ packages in R^35,58,59^. Models included population-level (fixed effects) and individual-level (random effects) parameters for all estimated models and sampled with weakly informative priors. To estimate chain convergence, we visually examined trace plots, pairs plots, and R-hat estimates and found them to show good chain convergence. We report the median, 89% CI, highest density interval (HDI), and probability of direction (pd) for parameters’ posterior distributions (logistic regression estimates are on the log-odds scale; for prior robustness checks, see Supplementary Information). Note that 89% CI was selected following recent recommendations for Bayesian posterior estimates^60^. Specifically, the 89% CI bares no special meaning, and is as arbitrary as other estimates (i.e., 95%). Instead of using the CI as a single estimate for drawing conclusions regarding the effect of interest, we made an effort to describe the full posterior (including explicit figures) and provide more information regarding a range of CIs where the conclusion of the analysis was not clear cut. All models were estimated by comparing Expected Log-Probability Density (i.e., elpd). For computational models, we performed model comparison using a leave-one-block-out approach. For each model, we left out one of the six blocks and calculated the expected log predictive density for each trial in the left-out block [i.e., we estimated the population and individual parameter posteriors using hierarchical Bayesian modeling (using ‘stan’ Markov chain Monte Carlo) and then calculated the log predictive density for each trial in the left-out block]. This procedure was repeated for all blocks resulting in pointwise predictions for each posterior sample and across all observations (that is, an estimated elpd for each observation). We then used the ‘loo_compare’ function (from the ‘loo’ package) to perform pairwise model comparisons between each model and the model with the largest elpd. An elpd difference of 2 times the standard error was considered substantial^35^. We further conducted posterior predictive checks to establish the conclusions arising from our model comparison results (Supplementary Information).

### Reporting summary

Further information on research design is available in the Nature Research Reporting Summary linked to this article.

### Supplementary information

Supplementary Information

Reporting summary

## Data Availability

All the data gathered and analyzed during this study are publicly available on https://osf.io/eyh7t/.

## Code availability

Regression analyses, computational modeling analysis codes and Supplementary Information are publicly available on https://osf.io/eyh7t/.
