# Supplementary material for: Disentangling the contribution of individual and social learning processes in human advice-taking behavior
Source: NPJ Sci Learn. 2024 Jan 20;9:4. doi: 10.1038/s41539-024-00214-0 (PMC10799906; doi:10.1038/s41539-024-00214-0)
Supplement: Supplementary file 1 — Supplementary Information [file 41539_2024_214_MOESM1_ESM.pdf]

Supplementary Information

Supplementary Methods Figures

Constructing the cards' expected values and teachers' accuracy. The likelihood of each card leading to a reward, and of the teacher choosing the card with the higher chances to gain reward, changed throughout the duration of the experiment. The reward probability and the advice accuracy followed a stochastic distribution (as shown in Supplementary Figures 1 and 2).

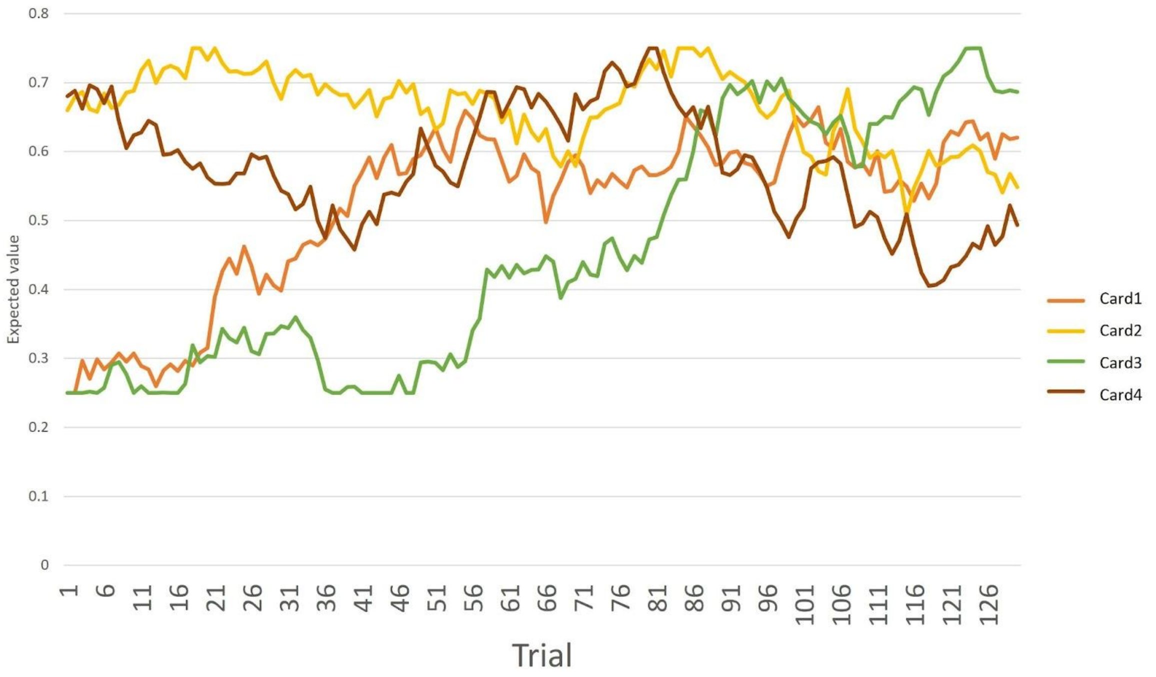

Supplementary Figure 1. Drifting expected values for the cards across trials. The same random walk was used in each of the 6 blocks, and each card was randomly assigned to a random walk.

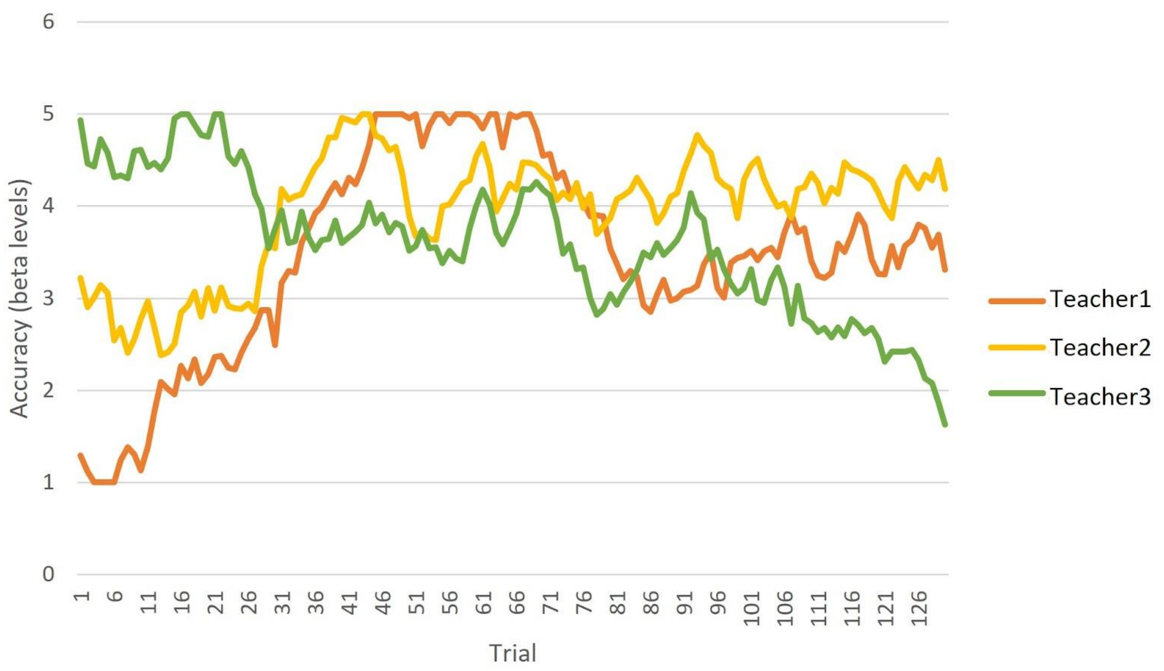

Supplementary Figure 2. Drifting accuracy rates for advice accuracy of three teachers. The same random walk was employed in every session, such that the accuracy rate for each teacher avatar was randomly assigned.

Supplementary Results

Computational modeling

Model 2b (dynamic non-informed advice-taking): While Model 2 (fixed non-informed advice taking) aimed to test a general tendency to follow advice, it does not specifically estimate the influence of choice difficulty on following advice. Therefore, Model 2b (dynamic non-informed advice taking) was constructed to formally elaborate this process. Here, we augmented Model 2 so that on trials in which the participants' decision was more difficult (the offered cards' internal values were similar), non-informed advice-taking tendencies were increased to further rely on external information. Specifically, this model assumes that the bias in Equation (4) changes as a function of the difference between the Q-values of the two cards that are currently offered to the player, resulting in Supplementary Equation 1:

(1) $\varphi = \varphi intercept + \varphi slope * |\Delta Qcards|$

φintercept serves as a general bias factor, and φslope serves as an additional slope parameter that is multiplied by the absolute difference between Qadvised_card and Qunadvised_card.

This model includes four free parameters: α, β, and two bias parameters (φintercept and φslope) The model comparison results showed that the elpd difference from the winning model was -512.3 (se= 33.3), hence it performed significantly worse from the model involving informed and non-informed advice taking.

Regression analyses. In our analyes, we dummy coded the outcome of the previous offer (i.e., 0 vs. 1 for *rewarded* vs. *unrewarded*), and the advice condition (i.e., 0 vs.1 for *concealed advice* vs. *revealed advice*).

Priors robustness analysis. We used N(0,0.2) priors for all the effects described in the regression analysis section. We conducted a prior robustness analysis which indicated that the reported effects hold even when having narrower N(0,0.1) or wider N(0,0.4) priors for our predictors.

Parameter recovery. To ensure that our model was well specified, we simulated 200 agents using the full model (individual learning, informed and non-informed advice-taking). Individual parameters for each agent were sampled hierarchically. Learning rates were sampled from a normal distribution with a mean of 0.5 and a SD of 1, and were constrained between 0 and 1 using a logit link function. Decision noise parameters were sampled from a normal distribution with a mean of 2 and a SD of 0.5, and constrained to be positive using an exponential link function. Omega parameters were sampled from a normal distribution with a mean of 0.5 and a SD of 1, and were constrained between 0 and 1 using a logit link function. Bias parameters rates were sampled from a normal distribution with a mean of 0.75 and a SD of 1, and were constrained between -1 and 1 using a scaled logit link function. For each agent, we sampled 6 blocks, of 130 trials each, using the same random walk that was used for the empirical data collection. All other task aspects were exactly the same as the empirical task. We then sampled posterior parameter distributions using four chains with 1000 warmup iteration and 250 posterior samples each, leading to a total of 1000 posterior samples over 156000 artificial observations. We then inspected both the population and individual-level parameters against the true parameter estimates used to simulate behavior. We found an excellent recovery, with a full recovery of all parameters (see Supplementary Figure 3).

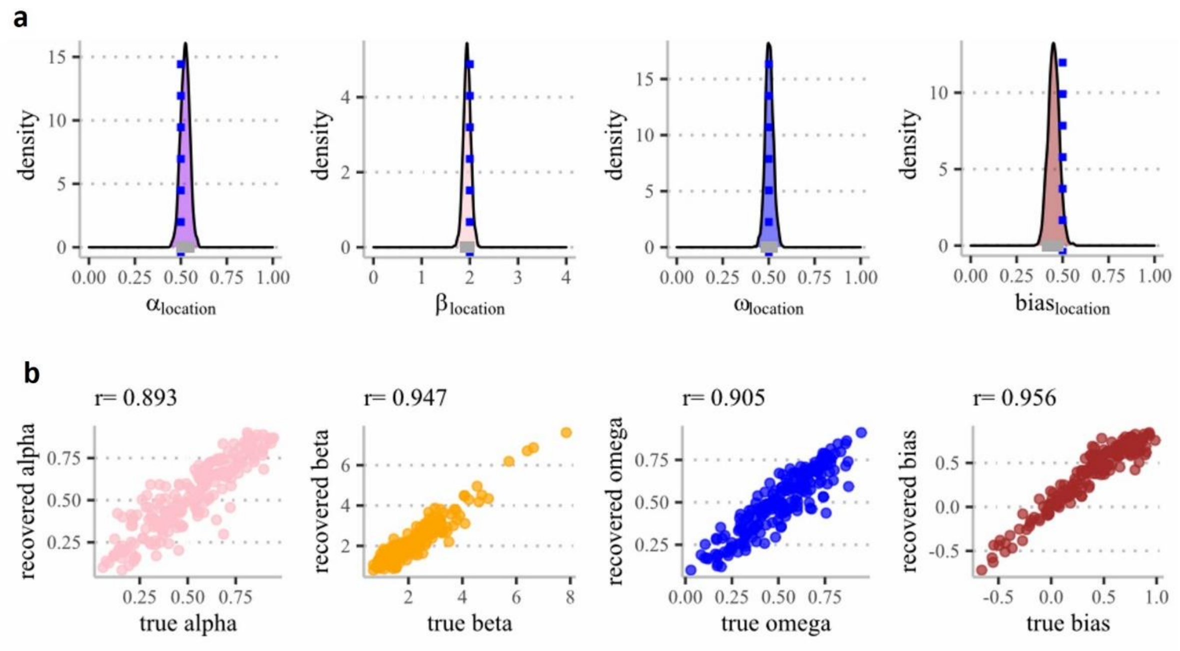

Supplementary Figure 3. Parameter recovery for the full model. Model parameters included alpha (learning rate), beta (decision temperature), omega (informed advice-taking) and bias (non-informed advice-taking). We simulated 200 agents using a hierarchical parameter sample and estimated the parameter from simulated behavior. **(a)** Population-level parameters – dashed blue lines indicate the true parameter estimates used for behavior simulations. Parameters estimation from simulated data is indicated using the posterior distributions. **(b)** Individual-level parameters – we averaged to posterior distribution for each individual at each parameter, and plotted these recovered estimates against the true parameters. Overall, we found excellent recovery for population and individual-level parameters.

How accurate is the model in estimating the quality of the teacher?

In the current design, the teacher's accuracy drifted slowly across trials (see Supplementary Figure 2). As a sanity check, we wanted to demonstrate that informed value learning reflected (at least to some extent) the accuracy of the teacher in a given trial. In order to test this hypothesis, we first used the winning model to estimate internal ΔQ-values for following advice (i.e., Q_{follow_advice} - Q_{reject_advice}, see Equations (7) and (8)) for each participant in each trial. We then performed a Bayesian logistic regression analysis to predict the internal values to follow advice (ΔQ_{follow_advice}, see Equation (8)) as a function of advice accuracy (teacher beta values, see Supplementary Figure 2). The results supported our hypothesis and demonstrated that advice accuracy predicted ΔQ_{follow_advice} (see Supplementary Figure 4; median = 0.02, CI95% = 0.02 to 0.02, pd~100%). Overall, this suggests that the model's latent values (that were based on empirical choice behavior) reflected teacher's accuracy to some extent, so that higher advice accuracy reflected in a higher tendency to follow advice.

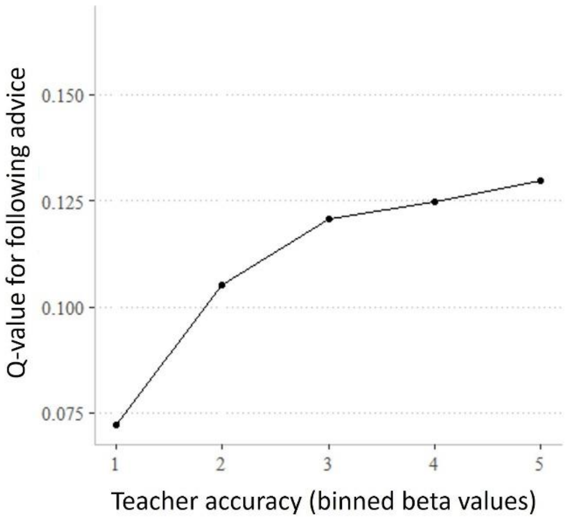

Supplementary Figure 4. Association between teacher's accuracy and empirical Q-values for following advice. Results of a regression analysis showing that the internal values for following advice (as estimated by the winning model) increased with teacher's accuracy. Across the task, the artificial teacher had access to the true expected values of the cards. The teacher chose a card according to a softmax with beta values that drifted slowly across each block (see Supplementary Figure 2). X-axis indicated binned teacher beta values (lower values suggesting less accurate advice). Y-axis indicates the internal value for following advice (i.e., difference between Q_{follow_advice} and Q_{reject_advice} estimated in the winning model). Overall, we found higher internal values for following advice when the teacher's accuracy was higher.

Non-social control study

In our student-teacher paradigm, we used a symbolic social manipulation, where participants were presented with a virtual teacher. However, the reveal effect that was described (increased tendency to choose the advised card in trials where the advice was revealed vs. concealed), might have been caused by non-social aspects of the task. To control for this explanation, we performed an additional study where the virtual teacher was replaced by a non-social lottery wheel. Here, a lottery wheel served as an external signal, indicating to participants which is the better choice (see Supplementary Figure 5). It is important to note that we kept all aspects exactly the same between the non-social and social studies, including the overall accuracy of the lottery wheel/virtual teacher in indicating the better choice across trials. The only difference was the framing, thus allowing us to estimate the influence of the symbolic social frame in the main study. We hypothesized that if indeed participants perceived the advice of the virtual teacher as social, we should observe a larger reveal effect for a virtual teacher compared with a lottery wheel.

Participants. 30 participants performed an online experiment (one participant did not complete the experiment, and two participants had over 25% outlier response times, and were removed from the analyses). Participants received payment for their participation in the experiment (50 NIS, approximately 13$).

Procedure. The procedure was very similar to that of the student-teacher paradigm described in the main text. The main difference was that the virtual teachers were replaced by a lottery wheel (Supplementary Figure 5, panel a). Participants were told that on some of the trials the outcome of a lottery will appear and indicate which of the cards was chosen. To correspond with the accuracy rates of the advice in the main study the instructions further mentioned that "many times the results of the lottery indicate the best card of the two, so that you can use this information if you wish. However, since this is a lottery, there could be times where the card with the lower value could be drawn." We also stated to participants that "the chance that the best card will be drawn in the lottery could change during the experiment" and that "the decision whether to use this information is your personal choice". All other aspects were exactly as in the study reported in the main text.

Results. We aggregated the observation from the current study (non-social manipulation using lottery wheel) with the observations from the study in the main text (symbolic social manipulation using a virtual teacher). We then performed a Bayesian regression analysis with Reveal (concealed vs. revealed advice), Study (social vs. non-social) and their paired interaction, predicting the coherence rate (the proportion of trials where the participant chose

the same card as the advisor). We found a substantial paired interaction, suggesting a greater casual influence of advice (i.e., greater reveal effect) on the symbolic social vs. non-social study (Supplementary Figure 5; posterior medium for the interaction between the studies was +0.11, HDI 89%=0.02 to 0.20 on the log-scale, pd = 96.96%). When performing simple effect comparison we find no evidence for a difference between social and non-social trials when advice is concealed (posterior medium for the difference was -.01, HDI 89%=-.09 to 0.05 on the log-scale, pd = 62.48%). This is expected since in both studies the conceal trials are identical. However, we did find higher coherence in the social vs. non-social study in the reveal trials, suggesting a larger causal influence of advice on choice behavior in the social framing (posterior medium for the difference was .10, HDI 89%=+0.03 to +0.16 on the log-scale, pd = 99.08%).These results suggest that there is an additional value for advice that is symbolically framed as social information.

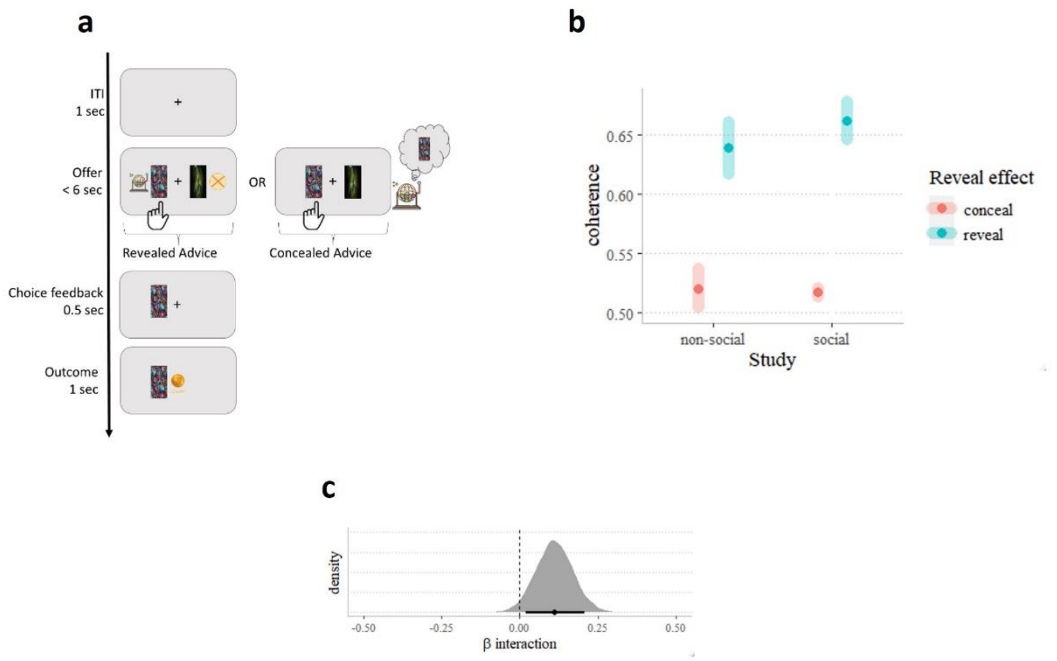

Supplementary Figure 5. Results for the social vs. non-social manipulation on the advice reveal effect. (**a**) Trial sequence for the non-social study where a lottery wheel was used instead of a virtual teacher. (**b**) The effect of the Social study (virtual teacher, main text) and the Non-social study (lottery wheel). The results show very similar coherence rates when advice was concealed, which is expected since these trials were the same in both studies. Importantly, we found lower coherence rates when the advice was revealed in the non-social compared with the social study. (**c**) Posterior distribution for the Study X Reveal effect (line indicated 89% HDI; estimates are on the log-scale, values suggest the amount of change in the reveal effect when the predictor changes from non-social to social).
